# Forgiveness of others and subsequent health and well-being in mid-life: a longitudinal study on female nurses

**DOI:** 10.1186/s40359-020-00470-w

**Published:** 2020-10-01

**Authors:** Katelyn N. G. Long, Everett L. Worthington, Tyler J. VanderWeele, Ying Chen

**Affiliations:** 1 Human Flourishing Program, Harvard Institute for Quantitative Social Science, 129 Mt Auburn Street, Cambridge, MA 02138 USA; 2 Department of Epidemiology, Harvard T.H. Chan School of Public Health, Boston, MA USA; 3 Department of Psychology, Virginia Commonwealth University, Richmond, VA USA; 4 Department of Biostatistics, Harvard T.H. Chan School of Public Health, Boston, MA USA

**Keywords:** Forgiveness, Health, Well-being, Outcome-wide epidemiology, Longitudinal, Mid-life

## Abstract

**Background:**

Forgiveness is a concept of growing interest within psychology and of potential relevance to public health. While there has been increasing evidence suggesting positive associations between forgiveness of others and a range of psychosocial well-being and mental health outcomes, its associations with health behaviors and physical health are less clear.

**Methods:**

This study used longitudinal data from the Nurses’ Health Study II (2008 Trauma Exposure and Post-traumatic Stress Supplementary Survey to 2015 questionnaire wave, *N* = 54,703), to conduct an outcome-wide analysis among a cohort of female nurses in the United States (age range: 43–64 years). The study prospectively examines the association between spiritually motivated forgiveness of others and a number of of subsequent psychosocial well-being, mental health, health behavior, and physical health outcomes in midlife. A set of linear, logistic, and Poisson regression models were used to regress each outcome on forgiveness in separate models. Sociodemographic factors, prior religious service attendance, and prior values of all outcome variables were controlled for wherever data were available. To account for multiple testing, we performed Bonferroni correction.

**Results:**

Forgiveness was associated with subsequent improved psychosocial well-being and reduced psychological distress outcomes in a monotonic pattern. For instance, the top versus bottom level of forgiveness was associated with substantially higher levels of subsequent positive affect (β = 0.18, 95% CI: 0.15, 0.21) and social integration (β = 0.15, 95% CI: 0.13, 0.17), and was inversely associated with several indicators of subsequent psychological distress such as depressive symptoms (β = − 0.16, 95% CI: − 0.19, − 0.14). However, in this sample, there was little evidence that forgiveness was associated with health behaviors or physical health outcomes.

**Discussion:**

This study suggests that forgiveness may be a health asset for promoting population mental health and psychosocial well-being, and moreover may also be understood as a good in itself. Further investigation on the dynamics between forgiveness and physical health is warranted to explore the discrepancy between the results here and some past research.

## Background

On the heels of devastating global events in the mid-twentieth century, philosophers, theologians, and social scientists grappled anew with the concept of forgiveness; its history, its definition, its mortality, and its impact [1]. Psychologists in particular, wanted to better understand the processes of forgiveness and its impact on psychosocial health and well-being, especially for those who had experienced significant personal trauma [2]. These efforts accelerated throughout the 1980s and 1990s, bolstered by external funding and increasingly robust empirical evidence, and eventually emerged as subfield that continues to grow across disciplinary lines [3]. While a single agreed-upon empirical definition of forgiveness remains somewhat elusive, a common understanding of forgiveness is as a prosocial behavior in which a victim replaces ill will toward the wrongdoer with goodwill, or, reducing negative thoughts, emotions, and behaviors and replacing these with positive thoughts, emotions, and behaviors towards the offender [4–6]. Forgiveness is likely shaped by a variety of factors, including an individual’s religious or spiritual beliefs [1]. Importantly, forgiveness is widely recognized as distinct from pardoning, condoning, excusing, justifying, and reconciling [2].

Empirically, the majority of prior observational studies on forgiveness and health have been carried out using cross-sectional data, from which causality cannot be inferred [7]. More rigorous evidence from the limited number of prior longitudinal studies and randomized trials suggests that forgiveness is associated with a number of psychosocial well-being and mental health outcomes such as lower levels of depression, anxiety, and hostility, higher positive emotion, higher satisfaction with life, and greater social support [7–9]. There is also evidence suggesting that forgiveness is associated with reduced nicotine dependence and substance abuse, and fewer self-reported physical illness symptoms [4]. Other researchers have hypothesized that forgiveness may confer health benefits through direct mechanisms such as promoting emotional regulation [10, 11] as well as through indirect mechanisms such as enhancing social integration [12]. However, the association between forgiveness and other health behaviors and objective measures of physical health outcomes remain unclear [13, 14]. While many of these outcomes are important domains for public health research and practice, the topic of forgiveness remains largely unaddressed within population health literature [5].

As interdisciplinary inquiries around forgiveness continue, calls have been made to address a number of knowledge gaps in the empirical evidence on forgiveness and health [3–5]. These include using longitudinal data to provide stronger evidence for inferring causal associations; considering a wider range of health and well-being outcomes, especially health behavior and physical health outcomes; understanding the dynamics of forgiveness and health at different stages of life; and considering more rigorous control for potential confounding and reverse causation by baseline health status [3, 4]. To address some of these knowledge gaps, this study used data from a well-established longitudinal cohort of female nurses in the U.S. to prospectively examine the association between spiritually motivated forgiveness of others and a wide range of subsequent psychosocial well-being, psychological distress, health behavior and physical health outcomes in mid-life, controlling for a wide array of participant baseline characteristics.

## Methods

### Study population

Data in this study comes from the Nurses’ Health Study II (NHSII) [15]. The NHSII cohort started in 1989 and enrolled 116,429 female registered nurses, ages 25–42 years at enrollment, living across 14 US states [16]. Every 2 two years since, NHSII participants have completed self-administered surveys by mail or online, with over 90% response rate at each follow-up cycle. The NHSII surveys ask respondents about numerous topics related to their psychosocial characteristics, health behaviors, and physical health [16]. Self-reported physical illness diagnoses are verified using medical records. A supplemental survey on Exposure and Post-Traumatic Stress was administered in 2008 in which several questions on spiritually motivated forgiveness were included. The analytic sample for the present study was drawn from participants of the 2008 supplementary survey, and we considered this year as the baseline for our study. Outcome variable data were drawn from the most recent NHSII questionnaire wave in 2015, however, if the outcome was not measured in 2015, we used data from earlier waves in 2013 or 2011 waves. Our total analytic sample was 54,703 participants for this study. Details regarding the sample derivation process are reported in the Supplementary Text and Figure. Further information about the Nurses’ Health Study is available on the website (https://www.nurseshealthstudy.org/). This study was approved by the Institutional Review Board at Brigham and Women’s Hospital.

### Measures

#### Forgiveness

Questions on spiritually-motivated forgiveness in the 2008 NHSII supplementary survey were taken from the Brief Multidimensional Measure of Religiousness/Spirituality [17], including, “Because of my spiritual or religious beliefs, I have forgiven those who hurt me”, which we treated as our main exposure. Answers were provided using a four-point scale: *always or almost always*, *often*, *seldom*, and *never*. In our analysis, “never” and “seldom” were collapsed due to data sparsity, yielding the following three response categories: never/seldom, often, always or almost always. For our extended analysis on self-forgiveness and divine forgiveness, conducted from the same sample, please see Long et al., 2020 [18].

#### Outcomes

Nineteen outcomes were assessed, using data from the 2011, 2013, or 2015 NHSII waves. Outcomes fell into four categories: (1) psychological well-being (i.e., positive affect and social integration); (2) psychological distress (i.e., depression diagnosis, depressive symptoms, anxiety symptoms, anxiety diagnosis, hopelessness, and loneliness); (3) health behaviors (i.e., heavy drinking, current cigarette smoking, frequent physical activity, preventive healthcare use, dietary quality); and (4) physical health (i.e., all-cause mortality, type 2 diabetes, stroke, heart diseases, cancer, overweight/obesity, number of physical health problems [sum of the above 5 physical illness conditions]). For more information about how each variable was assessed, please see our supplementary text.

#### Covariates

Covariate data were taken from the 2008 supplementary survey or prior questionnaire waves. Covariates included the following: age (in years), race/ethnicity (non-Hispanic white, others), geographic region (Northeast, South, West, Midwest), marital status (married/in domestic relationship, unmarried), pre-tax household income (<$50,000, $50,000-$74,999, $75,000-$99,999, ≥$100,000), census tract median income (<$50,000, $50,000-$74,999, $75,000-$99,999, ≥$100,000), employment status (currently employed, non-employed), subjective social standing in US and in community (both rated on a scale ranging from 1 to 10), census tract college education rate (continuous), night shift work over past two years (none, 1–9 months, 10–19 months, 20+ months), childhood abuse (a summary score 0 to 5 [19];), number of close friends (none, 1–2, 3–5, 6–9, 10 or more), religious service attendance (never/almost never, less than once/week, at least once/week), menopausal status (premenopausal or uncertain, postmenopausal), and post-menopausal hormone use (yes, no). To reduce the possibility of reverse causation [20], wherever data were available, we controlled for prior values of all outcome variables. These included the prior values of: positive affect, depression diagnosis, prior depressive symptoms, phobic anxiety, hopelessness, alcohol intake, smoking status, physical activity, preventive healthcare use, AHEI dietary score, history of type 2 diabetes, stroke, heart disease, or cancer, and body mass index. In a sensitivity analysis, we also considered history of trauma exposure (yes, no) as a covariate. Trauma exposure in this analysis was defined as traumatic events which likely involved offenses inflicted by other people (e.g., rape, physical abuse), measured with a modified version of the Brief Trauma Interview [21].

### Statistical analysis

All statistical analyses were performed in SAS 9.4. We examined participant baseline characteristics across levels of forgiveness using the analysis of variance and Chi-square tests.

The primary analyses employed an outcome-wide analytic approach [20, 22] using a set of regression models to regress each outcome variable on forgiveness separately. Depending on the distribution of the outcome variable, one of the following models was run: (1) linear regression models for continuous outcomes; (2) logistic regression models for binary outcomes with a prevalence < 10%; (3) Poisson regression models for binary outcomes with a prevalence ≥10% [23, 24]. All models were fully adjusted for all covariates simultaneously. We standardized all continuous outcome variables (mean = 0, standard deviation = 1), which allowed the effect estimates to be reported in terms of standard deviation change in the outcome variable, and also allowed us to better compare effect estimates across all outcomes. To account for multiple testing, we performed Bonferroni correction.

#### Multiple imputation

To impute missing data on all variables, we used multiple imputation by chained equations (with five imputed datasets). Compared to other methods of addressing missing data, this method often offers greater flexibility in missing data patterns in order to produce less biased estimates [25–27].

#### Sensitivity analyses

Several sensitivity analyses were conducted. First, we calculated E-values to evaluate robustness of the associations to potential unmeasured confounding [28, 29]. Specifically, E-values assess the minimum strength that an unmeasured confounder would have to have on the risk ratio scale with both the exposure (forgiveness) and the outcome to explain away the association. Second, we conducted a complete-case analysis restricting the analysis to participants with complete data to compare results with our primary analysis that used imputation procedures to account for missing data. Third, to better understand the relationship between forgiveness and first-time occurrence, or *incidence,* of physical health problems, we reanalyzed models of each physical health outcome, one at a time, not including those with a particular condition at baseline. For example, to examine the relationship between forgiveness and incident heart disease, we ran the analysis with only those who had *not* been diagnosed with heart disease at baseline in order to assess first-time diagnosis of heart disease at later waves. Finally, we reanalyzed the primary sets of models stratified by participant history of exposure to trauma that likely involved offenses inflicted by other people and for which forgiveness of others may be relevant.

## Results

### Descriptive analyses

In Table 1, we show baseline participant characteristics by forgiveness, and in Table S1, we show participant characteristics for the full sample as well as the timing of assessment for all study variables. The average age of respondents was 53.37 years old (Standard Deviation (SD) = 4.65). The participants were primarily non-Hispanic White (95.75%), currently employed (88.78%), married (81.41%), and with nearly half (49%) reporting an income between $50,000–$74,999 (see Table S1). The majority of participants reported a high level of forgiveness (52.73% reported often, and 36.58% reported always/almost always). Participants who were married, attended religious services often, and had lower level of psychological distress and healthier behaviors were more likely to report a high level of forgiveness (Table 1).

**Table 1:** Participant characteristics by forgiveness of others at study baseline (The Nurses’ Health Study II 2008 Supplementary Survey, *N* = 53,237)^a^

Participant characteristics	Forgiveness of others		
	Never/seldom (*n* = 5692)	Often (*n* = 28,071)	Always/almost always (*n* = 19,474)
Age, in years (range: 43–64)^b^	53.26 (4.62)	53.29 (4.65)	53.46 (4.66)
Non-Hispanic white, %	95.06	95.95	95.62
Marital status, %	76.10	81.07	83.57
Geographic region, %			
- Northeast	36.84	33.45	28.13
- Midwest	28.91	32.76	34.85
- South	15.76	18.05	21.14
- West	18.48	15.74	15.88
Subjective SES in US (range: 1–10)	6.92 (1.41)	7.06 (1.29)	7.22 (1.31)
Subjective SES in community (range: 1–10)	6.59 (1.70)	6.86 (1.56)	7.12 (1.54)
Census-tract college education rate (range: 0–0.88)	0.33 (0.17)	0.32 (0.16)	0.31 (0.16)
Household income, %			
-< $50‚000	17.32	15.66	15.66
- $50‚000-$74‚999	27.14	27.34	27.78
- $75‚000-$99‚999	19.54	21.70	21.69
- > =$100‚000	35.99	35.31	34.87
Census tract median income, %			
- < $50‚000	23.33	25.29	27.49
- $50‚000-$74‚999	47.54	49.15	49.38
- $75‚000-$99‚999	20.85	19.27	17.87
- > =$100‚000	8.29	6.29	5.26
Night shift work over past 2 years, %			
- none	90.70	92.05	92.44
- 1-9 months	4.04	3.45	3.18
- 10-19 months	1.40	1.29	1.29
- 20+ months	3.85	3.20	3.09
Currently employed, %	89.19	89.64	87.41
Childhood abuse victimization (range: 0–5)	2.06 (1.57)	1.77 (1.48)	1.65 (1.48)
Religious service attendance, %			
- never/almost never	42.28	22.70	16.42
- < once/week	40.10	39.82	29.83
- > =once/week	17.62	37.48	53.75
Number of close friends (range: 0–5)	1.55 (0.70)	1.71 (0.65)	1.80 (0.65)
Depressive symptoms (range: 0–30)	8.57 (5.96)	6.40 (4.92)	4.82 (4.43)
Depression diagnosis, %	20.85	15.39	13.17
Anxiety symptoms (range: 0–15)	3.05 (2.57)	2.55 (2.23)	2.10 (2.05)
Hopelessness (range: 0–3)	1.22 (0.94)	0.91 (0.89)	0.69 (0.91)
Positive affect (range: 0–3)	1.77 (0.82)	2.04 (0.74)	2.27 (0.74)
Preventive healthcare use, %	82.01	85.60	85.82
Alcohol intake, %			
- 0 g/day	31.14	31.31	38.20
- 0.1–9.9 g/day	44.45	46.40	41.77
- 10.0-29.9 g/day	18.45	18.42	16.46
- 30+ grams/day	5.96	3.88	3.58
Cigarette smoking, %			
- never smoker	59.73	65.27	69.06
- former smoker	32.03	28.58	25.71
- current smoker 1–14/day	4.14	3.45	2.98
- current smoker 15–24/day	2.97	1.99	1.75
- current smoker > = 25/day	1.12	0.71	0.49
Physical activity (METS ^c^), %			
- < 3	20.37	15.56	15.36
- 3-8.9	18.85	19.23	18.78
- 9-17.9	19.64	20.84	20.69
- 18-26.9	12.31	13.98	14.64
-> =27	28.83	30.39	30.53
Dietary quality (AHEI score ^d^), %			
- bottom tertile, %	37.22	32.87	30.47
- middle tetile, %	32.72	33.75	33.08
- top tertile, %	30.06	33.38	36.45
BMI categories (kg/m^2^), %			
- < 20	5.33	5.30	5.63
- 20-24.9	35.35	37.54	39.40
- 25-29.9	28.87	29.71	29.63
- 30-34.9	16.42	15.52	14.40
- 35+	14.03	11.93	10.94
Diabetes, %	5.52	4.63	4.41
CHD, %	1.13	1.14	1.16
Stroke, %	1.45	1.32	1.31
Cancer, %	6.35	6.52	6.64
Postmenopausal status, %	59.90	60.68	60.61
Replacement Hormone use, %	14.31	14.15	14.85

Values are means (SD) for continuous variables and percentages for categorical variables and are standardized to the age distribution of the study population. Values of polytomous variables may not sum to 100% due to rounding

^a^ This table is based on participants with complete data on forgiveness

^b^ Value is not age adjusted

^c^ Metabolic equivalents score (METS) was used to measure physical activity

^d^ Alternate Healthy Eating Index (AHEI) was used to measure dietary quality

### Forgiveness and subsequent health and well-being

In Table 2, we report the main outcome-wide analysis on spiritually motivated forgiveness and subsequent health and well-being outcomes. Forgiveness was positively associated with subsequent psychosocial well-being and inversely associated with subsequent psychological distress outcomes in a monotonic pattern. For instance, those who forgave most frequently (versus those who never or seldom forgave) reported substantially higher levels of positive affect (Beta Coefficient (β) = 0.18, 95% Confidence Interval (CI): 0.15, 0.21) and social integration (β = 0.15, 95% CI: 0.13, 0.17), and had substantially lower levels of subsequent depressive symptoms, anxiety symptoms, hopelessness and loneliness (e.g., β = − 0.16, 95% CI: − 0.19, − 0.14 for depressive symptoms; β = − 0.17, 95% CI: − 0.20, − 0.14 for hopelessness).

**Table 2:** Forgiveness of others and subsequent health and well-being in mid-life (The Nurses’ Health Study II 2008 supplementary survey to 2011, 2013 or 2015 questionnaire wave, *N* = 54,703^a^)

	Forgiveness of others ^b^							
	Often vs. Never/seldom				Always/almost always vs. Never/seldom			
Health and well-being outcomes	RR^c^	β^d^	95% CI	*P*-value threshold	RR^c^	β^d^	95% CI	*P*-value threshold
**Psychosocial Well-being**								
Positive affect		0.09	0.06, 0.12	<.0026^e^		0.18	0.15, 0.21	<.0026^e^
Social integration		0.09	0.06, 0.11	<.0026^e^		0.15	0.13, 0.17	<.0026^e^
**Psychological Distress**								
Depression diagnosis	0.94		0.87, 1.02		0.91		0.83, 1.00	
Depressive symptoms		−0.09	−0.12, −0.07	<.0026^e^		−0.16	−0.19, −0.14	<.0026^e^
Anxiety symptoms		0.00	−0.03, 0.03			−0.11	−0.14, −0.08	<.0026^e^
Hopelessness		−0.10	−0.13, − 0.08	<.0026^e^		− 0.17	− 0.20, − 0.14	<.0026^e^
Loneliness		− 0.08	− 0.11, − 0.05	<.0026^e^		− 0.12	− 0.15, − 0.09	<.0026^e^
**Health Behaviors**								
Heavy drinking	1.00		0.84, 1.20		1.00		0.80, 1.25	
Current cigarette smoking	1.00		0.83, 1.21		0.99		0.81, 1.21	
Frequent physical activity	1.00		0.97, 1.04		1.01		0.97, 1.05	
Preventive healthcare use	1.01		0.98, 1.04		1.00		0.97, 1.04	
Dietary quality		0.01	−0.01, 0.04			0.04	0.01, 0.07	<.01
**Physical Health**								
All-cause mortality	1.03		0.80, 1.32		1.17		0.90, 1.53	
No. of physical health problems		0.00	−0.02, 0.02			− 0.01	− 0.03, 0.01	
Diabetes	0.98		0.85, 1.14		0.95		0.81, 1.12	
Stroke	1.12		0.81, 1.56		1.11		0.78, 1.59	
Heart Disease	0.86		0.57, 1.29		1.15		0.74, 1.78	
Cancer	0.97		0.89, 1.06		0.99		0.90, 1.08	
Overweight/obesity	1.01		0.97, 1.05		0.99		0.95, 1.03	

*Abbreviations*: *RR* risk ratio, *CI* confidence interval

^a^ The full analytic sample was restricted to those who responded to the Nurses’ Health Study II 2008 supplementary survey in which the exposure variable forgiveness was assessed. Multiple imputation was performed to impute missing data on all variables

^b^ A set of generalized estimating equations were used to regress each outcome on forgiveness separately. All models controlled for participants’ age, race, marital status, geographic region, childhood abuse, socioeconomic status (subjective SES, household income, census tract college education rate, and census tract median income), employment status, night shift work schedule, religious service attendance, number of close friends, prior health status or health behaviors (prior depressive symptoms, depression diagnosis, anxiety symptoms, hopelessness, positive affect, dietary quality, body mass index, smoking, alcohol intake, physical activity, preventive healthcare use, postmenopausal status, menopausal hormone therapy use, history of diabetes, heart diseases, stroke, and cancer)

^c^ The effect estimates for the outcomes of heavy drinking, current smoking, mortality, diabetes, heart diseases, stroke and cancer were odds ratio. These outcomes were rare [prevalence< 10%], so the odds ratio would approximate RR. Effect estimates for other dichotomized outcomes were RR

^d^ All continuous outcomes were standardized (mean = 0, standard deviation = 1), and β was the standardized effect size

^e^ *p* < 0.05 after Bonferroni correction (the *p* value cutoff for Bonferroni correction is *p* = 0.05/19 outcomes = 0.0026)

There was, however, little evidence of association between forgiveness and subsequent health behaviors and physical health outcomes, with the exception of higher dietary quality for those who reported frequent forgiveness compared to those that seldom or never forgave (β = 0.04, 95% CI: 0.01, 0.07), though this association did not pass the *p* < .05 threshold after Bonferroni correction for multiple testing.

### Sensitivity analysis

The E-values suggested that associations between forgiveness and psychosocial outcomes were at least somewhat robust to potential unmeasured confounding (Table 3). For example, an unmeasured confounder that was associated with both forgiveness and positive affect by risk ratios of 1.64, beyond all covariates the analysis already adjusts for, could serve to explain away the observed association, but weaker confounding could not. Similarly, to shift the confidence interval to include the null, an unmeasured confounder would need to be associated with both forgiveness and positive affect by risk ratios of 1.56, however weaker levels of confounding could not. In comparison, there was little evidence that the associations of forgiveness with health behaviors or physical health outcomes were robust to potential unmeasured confounding (Table 3).

**Table 3:** Robustness to unmeasured confounding (E-values ^a^) for assessing the associations between forgiveness and health and well-being (The Nurses’ Health Study II 2008 Supplementary Survey to 2011, 2013 or 2015 Questionnaire Wave)

Health and well-being outcomes	Forgiveness of others (always/almost always vs. never/seldom)	
	For effect estimate ^b^	For CI limit ^c^
Positive affect	1.64	1.56
Social integration	1.56	1.50
Depression diagnosis	1.43	1.00
Depressive symptoms	1.58	1.52
Anxiety symptoms	1.45	1.36
Hopelessness	1.61	1.53
Loneliness	1.47	1.39
Heavy drinking	1.00	1.00
Current cigarette smoking	1.11	1.00
Frequent physical activity	1.01	1.00
Preventive healthcare use	1.00	1.00
Dietary quality	1.23	1.12
All-cause mortality	1.17	1.00
No. of physical health problems	1.11	1.00
Diabetes	1.29	1.00
Stroke	1.11	1.00
Heart Disease	1.15	1.00
Cancer	1.11	1.00
Overweight/obesity	1.11	1.00

*Abbreviations*: *CI* confidence interval

^a^ See VanderWeele and Ding (2017) [29] for the formula and Mathur et al. (2018) [28] for the website and R package for calculating E-values

^b.^ The E-values for effect estimates are the minimum strength of association on the risk ratio scale that an unmeasured confounder would need to have with both the exposure and the outcome, above and beyond the measured covariates, to fully explain away the observed association of forgiveness (always/almost always vs. never/seldom) with various outcomes

^c.^ The E-values for the limit of the 95% confidence interval closest to the null denote the minimum strength of association on the risk ratio scale that an unmeasured confounder would need to have with both the exposure and the outcome, above and beyond the measured covariates, to shift the confidence interval to include the null value

The complete case analysis yielded very similar results to our main analysis (Table S2). The sensitivity analysis examining associations between forgiveness and incident physical health problems again suggested little evidence of association (Table S3). Finally, the analysis stratified by history of exposure to traumatic events found no substantive differences in associations of forgiveness across outcomes among those who had and had not been exposed to trauma (Table S4).

## Discussion

In 2019, film audiences around the world were captivated by *A Beautiful Day in the Neighborhood,* based on a true story of beloved children’s TV personality, Mr. Rogers, and his relationship with a journalist facing many personal struggles [30]. In the opening scene, Mr. Rogers greets his viewers and says, “Today, I’d like you to meet a new friend of mine named Lloyd Vogel.” He then carefully opens a carboard window to reveal a man with a bruised face. Quick to address the surprising image, Mr. Rogers says, “Someone has hurt my friend Lloyd, and not just on his face. He is having a hard time forgiving the person who hurt him. Do you know what it means, to forgive?” Here, he pauses to let the question sink in, and then says, “It is a decision we make to release a person from the feelings of anger we have at them. It’s strange, but sometimes it’s hardest of all to forgive someone we love” [31]. As the story unfolds, the audience learns more about the person who hurt Mr. Vogel and his difficult journey towards forgiveness, supported by friends and family, including Mr. Rogers. Illustrated so clearly in the film, Mr. Rogers’ widespread appeal was rooted in his paradoxical approach to children’s programming. Instead of focusing only on the innocence and playfulness of early years, he helped children name and deal with some of the most difficult aspects of life, often introducing equally complex and difficult ideas, like forgiveness, to help his young audience (and their families) cope with challenging circumstances.

Mr. Rogers was not alone in his curiosity about somewhat intangible aspects of human life, like forgiveness, and their real impact on health and well-being. As noted in the introduction, the empirical literature on this topic has expanded dramatically and the present study was intended to contribute yet further to this.

### Forgiveness, mental health, and psychosocial well-being

This study found strong positive associations between spiritually motivated forgiveness and psychosocial well-being and strong inverse associations with psychological distress outcomes. These findings are consistent with the large body of existing research on forgiveness, mental health, and well-being [32–34]. For example, a 2015 comprehensive review by Griffin et al. included 55 largely cross-sectional studies and used the stress-and-coping model as a paradigm to explore emotion-focused health outcomes that emerged from coping via either forgiveness or unforgiveness [32, 35]. Findings indicated that both state forgiveness (i.e., forgiveness of a specific offense) and trait forgiveness (i.e., a general disposition to forgive) were inversely related to psychological symptoms, with early evidence that older age, female sex, and enhanced motivation to forgive might serve as moderators [32]. Results in our longitudinal analysis of middle-aged female nurses align with these findings as greater forgiveness was inversely associated with multiple indicators of psychological distress in a monotonic pattern. A more recent review including a broader array of literature, found 714 published, peer-reviewed scientific journal articles on associations between forgiveness and general psychological adjustment (an integrated concept including well-being and mental health) between 1947 and 2018 [36]. Among these studies, the authors found extensive positive and consistent correlations between forgiveness and psychological adjustment, across numerous aspects of mental health or well-being. Despite the overwhelming trends however, the authors noted that the lack of sophisticated research designs limited statements on directionality and causality. The present analysis extends the literature by using longitudinal data and applying extensive control for potential confounding and reverse causation, which helps provide robust evidence.

### Forgiveness, health behaviors, and physical health

Critically, our analysis also included physical health and health behavior outcomes, finding little evidence of a longitudinal association over up to seven years, with the exception of some evidence for improved dietary quality among those who reported forgiveness most frequently. These findings initially seem to contradict a growing number of studies that find positive correlations between forgiveness and improved physical health and health behaviors. In their 2020 review of existing empirical research on forgiveness and physical health, Toussaint et al. note that despite the overwhelmingly positive correlations between forgiveness and self-reported health, reverse causation cannot be ruled out due to the lack of longitudinal design [37]. In other words, while forgiveness may result in better physical health, it may be the case that those in better physical health or with better health behaviors might be more likely to forgive. Evidence from the present study reinforce the need to address issues of reverse causality in determining association. However, relatively weak or near-null associations (Table 2) and very modest E-values for physical health and health behavior outcomes (Table 3) also support Toussaint et al.’s claim that “third variables” or confounders such as dispositions, biological factors, and other behaviors must be studied further in order to fully understand the relationship between forgiveness and physical health. While the present study controlled for a wide variety of social and economic factors, it is possible that there are indeed other factors that might interact with the relationship between forgiveness and physical health.

It is therefore fair to consider how the findings from this present study interact with findings of dozens of other (largely cross-sectional) studies that report positive correlations between forgiveness and physical health. Here we note four factors to be considered when weighing the cumulative evidence. First, in previous studies, physical health and health behaviors have most often been assessed using self-rated health, health-related quality of life, self-reported symptoms, and physical activity [7]. In contrast, the present study relied on medical records to verify diagnoses for physical illness outcomes, meaning that the “physical health” outcomes under examination in previous studies and the present study are somewhat distinct. Second, the health benefits of forgiveness may not be as substantial as the adverse health consequences of unforgiveness [7, 38]; our sample reported relatively high levels of forgiveness (nearly 90% reported forgiving often or more). Third, forgiveness interventions are often assessed among populations with poor health, in therapy, or those who have experienced a trauma, therefore studies that assess a larger, more general population with lower levels of trauma may find different associations with physical health. Finally, the present study had a limited follow-up period (up to seven years) among a relatively healthy sample, which may not have been enough time to assess the relationship between forgiveness and physical health, particularly chronic diseases which develop slowly over the life-course. Many researchers consider unforgiveness a stress-response to a transgression [39, 40]. Stress-related physical health outcomes tend to take a long time to develop. Whereas cross-sectional studies cannot indicate causal direction, they do suggest potential lifelong effects (in addition to those arising from reverse causation). This provides an additional reason for future research to investigate the forgiveness-health outcome relationship over longer longitudinal studies, with and without religious or spiritual qualification, and in more diverse contexts.

Further work to explore potential mechanisms is also needed. Recent studies, mostly cross-sectional, have suggested that rumination, anger [41], empathy [42], self-regulation [43], and emotional regulation [11] as possible mechanisms of forgiveness. Further studies on such mechanisms with longitudinal data and formal mediation analyses would be warranted.

### Limitations and strengths

First, the study was limited by a single item to assess religiously or spiritually motivated forgiveness of others, whereas a more generic assessment of forgiveness was not available. Second, the use of observational data introduces the potential for confounding due to unmeasured factors. We reduced concerns about this limitation through the use of prospective data, rigorous covariate control, and sensitivity analysis to assess unmeasured confounding. Next, the study included a largely homogenous sample of female nurses, limiting the applicability of findings to the general population. Lastly, there was no data on potentially important modifiers such as the severity of the offense or the reason or motivation for forgiveness.

The main strength of this study is the use of longitudinal data with a large cohort, which addresses a persistent gap in forgiveness research. We also reduced concerns about potential reverse causation by adjusting for prior values of all outcome variables, providing stronger evidence for causality [44–46]. By using the outcome-wide approach [20, 22], we were able to examine associations with multiple outcomes simultaneously, which, in turn, allowed us to report on strong associations as well as weak or null associations, findings that are often excluded in favor of publishing “significant” findings only. Finally, our supplementary analyses helped support our main analysis as each yielded similar resluts, which helped provide evidence that the results were robust to our modelling decisions.

### Implications

Findings from this study suggest that promoting forgiveness might be an innovative focus for public health interventions aimed at improving mental and psychosocial well-being. While forgiveness has not been historically included in public health discourse, public health researchers and practitioners around the world are increasingly turning attention towards determinants of health that fall outside traditional clinical and public health frameworks, for example the forthcoming Lancet commission on the emotional determinants of health [47]. Existing and widely studied forgiveness interventions such as Enright’s Process Model [48] and Worthington’s REACH Forgiveness model [40], have demonstrated their potential to improve forgiveness, reduce depression and anxiety, and increase hope [9, 49]. While many forgiveness interventions involve trained professionals, early evidence indicates that do-it-yourself forgiveness workbooks can effectively increase forgiveness [50], findings which are currently being tested in a randomized trial across five countries [51]. Importantly, any public health effort to promote forgiveness must be clear in what forgiveness is not; it is not foregoing justice, it is not excusing, or reconciling, or condoning. But it is, as Mr. Rogers’ character summarizes so clearly, releasing ourselves from feelings of anger, and replacing negative thoughts, emotions, or behaviors with positive ones. In some estimations, forgiveness can even be considered a species of love and a good in itself [52]. In a time of global pandemics, divisiveness, disparity, and uncertainty, perhaps the promotion of forgiveness would help support needed gains in our collective mental and social well-being.

## Conclusion

This study suggests that forgiveness is positively associated with multiple indicators of subsequent psychosocial and mental health. However, there was little evidence from the study of association with subsequent health behaviors or physical health outcomes. Forgiveness may also be understood as a good in itself. The associations reported here may have potential population health implications for promoting mental health and psychosocial well-being. The discrepancies between the weak associations with subsequent physical health in the present study, in contrast with much of the remainder of the literature, warrant further investigation to come to a better understanding of whether, and in what contexts, forgiveness of others might improve not only mental health and wellbeing, but physical health as well.

## Supplementary information

**Additional file 1.**

## Data Availability

The data of the NHSII study are not publicly available. Further information including the procedures to obtain and access data from NHSII is described at https://www.nurseshealthstudy.org/researchers (email: nhsaccess@channing.harvard.edu).

## Abbreviations

NHSII: Nurses’ Health Study II
SD: Standard Deviation
β: Beta Coefficient
CI: Confidence Interval
MA: Massachusetts
RR: Risk Ratio
USA: United States of America
VA: Virginia
