# Supplementary material for: Forgiveness of others and subsequent health and well-being in mid-life: a longitudinal study on female nurses
Source: BMC Psychol. 2020 Oct 1;8:104. doi: 10.1186/s40359-020-00470-w (PMC7528379; doi:10.1186/s40359-020-00470-w)
Supplement: Supplementary file 1 — Additional file 1. [file 40359_2020_470_MOESM1_ESM.docx]

**SUPPLEMENTARY TEXT**

**Outcome assessment**

*Positive affect.* To assess positive affect, “I was happy”, an item from the CESD-10 scale (1) was used, with responses ranging from 0 (rarely or none of the time) to 3 (all the time). Response to this question were treated as a continuous score, with higher scores representing greater positive affect.

*Social integration.* To measure social integration, the simplified Berkman-Syme Social Network Index was used (2). We included three self-reported aspects of social integration: 1) marital status (married, others), 2) number of close friends (none, 1-2, 3-9, 10+), and 3) participation in community, volunteer groups or other group organizations (none, 1-2 hours/week, 3-10 hours/week, and 11+ hours/week). In the original index, Religious service attendance was also included, however, we did not include it in our measure of social integration because it was already treated as an exposure variable in our study. Scoring criteria followed prior work (3). The overall score was derived (ranging from 0 to 9) by summing scores of the three dimensions.

*Depression.* Self-reported depression diagnosis was reported as yes or no. Additionally, the validated CESD-10 (1) was used to assess depressive symptoms over the past week with responses ranging from 0 (rarely or none of the time) to 3 (all of the time). When necessary, reverse coding was used so that higher scores indicated more depressive symptoms; overall scores were calculated using the sum across all items (α = 0.84, range=0 to 30).

*Anxiety symptoms.* To assess anxiety symptoms over the past four weeks, the 7-item Generalized Anxiety Disorder Scale was used (4). Response categories ranged from 0 (not at all) to 3 (nearly every day). Summary scores were created by summing responses across all items, with higher scores representing more anxiety symptoms (α = 0.88, range=0 to 21). The scale has been previously validated and demonstrated good psychometric properties (4).

*Hopelessness.* We used an item from the CESD-10 scale to assess hopelessness (1): “I felt hopeful about the future.”, with response options ranging from 0 (rarely or none of the time) to 3 (all of the time). Scores were reverse scored, such that hopelessness was indicated by higher scores.

*Loneliness.* We used an item from the CESD-10 scale (1) to assess loneliness: “I felt lonely.”, with responses ranging from 0 (rarely or none of the time) to 3 (all the time). Responses were treated as a continuous score, with higher scores indicating greater loneliness.

*Heavy drinking*. Heavy drinking was assessed by responses to the following question: “In a typical month during the past year, what was the largest number of drinks of beer, wine, and/or liquor you may have had in one day.” Responses ranged from 1 (none) to 6 (15 or more). Reporting more than five drinks in a single day was considered heavy drinking (5).

*Cigarette smoking.* Cigarette smoking was assessed with the question: “Do you currently smoke cigarettes?”. Reponses were either “yes” or “no”.

*Frequent physical activity.* Following prior of Hu et al., 1999 (6), frequent physical activity was assessed through validated questionnaires and a metabolic equivalent value (MET). MET scores ≥ 7.5 hours per week were treated as the minimum recommended level of physical activity (7).

*Preventive healthcare use.* Preventive healthcare use was assessed by asking participants if they had a physical exam for screening purposes in the past two years. Responses were either “yes” or “no”.

*Dietary quality.* To assess overall dietary quality, the Alternative-Healthy Eating Index (AHEI) was used (8) to calculate data from the food frequency questionnaire (FFQ). Better dietary quality was indicated by higher scores.

*All-cause mortality.* Regardless of cause, all deaths that took place by the end of the 2015 questionnaire wave were evaluated through reports from the national death index, state vital records, and next of kin.

*Number of physical health problems.* Participants self-reported if they had received a diagnosis for any of the following conditions: cancer, coronary heart disease, stroke, Type 2 diabetes. Self-reported diagnoses were validated by medical records. Additionally, self-reported height and weight were used to calculate BMI, and validated against objectively measured BMI in a subgroup of participants (r=0.97) (9). Overweight/obesity was considered as BMI≥ 25 kg/m2 (10). A summary score of number of physical health problems was created by summing the total of the following: overweight/obesity, cancer, coronary heart disease, stroke, and diabetes.

**Sample Derivation**

The sample for the present study was drawn from participants of the Nurses’ Health Study II (NHSII) 2008 Exposure and Post-Traumatic Stress Supplemental Survey (N=54,703), in which a question on spiritually or religiously motivated forgiveness of others was first included in the cohort; thus, this year was considered as the baseline for the present study. Data on the outcomes were taken from the most recent NHSII questionnaire waves, primarily the 2015 questionnaire; if the outcome was not assessed in the 2015 survey, we used outcome data from the 2013 or 2011 waves (see Supplementary Figure 1 below for a visual presentation). Among participants of the 2008 supplementary survey, 1,466 participants had missing data on forgiveness of others, another 17,168 participants had missing data on at least one covariate (all covariates had less than 6% of missing data, except for household income which had 15.7% of missing data), and the number of participants with missing data or were lost to follow-up on the outcomes variables ranged from 0 to 4,302 individuals, depending on the outcome. Multiple imputation was performed to impute missing data on all variables. This yielded an analytic sample of 54,703 participants for the analyses.

**REFERENCES FOR THE SUPPLEMENTARY TEXT**

1. Andresen EM, Malmgren JA, Carter WB, Patrick DL. Screening for depression in well older adults: evaluation of a short form of the CES-D (Center for Epidemiologic Studies Depression Scale). Am J Prev Med. 1994 Apr;10(2):77–84.

2. Berkman LF, Syme SL. Social networks, host resistance, and mortality: a nine-year follow-up study of Alameda County residents. Am J Epidemiol. 1979 Feb;109(2):186–204.

3. Chang S-C, Glymour M, Cornelis M, Walter S, Rimm EB, Tchetgen Tchetgen E, et al. Social Integration and Reduced Risk of Coronary Heart Disease in Women: The Role of Lifestyle Behaviors. Circ Res. 2017 Jun 9;120(12):1927–37.

4. Spitzer RL, Kroenke K, Williams JBW, Löwe B. A brief measure for assessing generalized anxiety disorder: the GAD-7. Arch Intern Med. 2006 May 22;166(10):1092–7.

5. Harrington LB, Hagan KA, Mukamal KJ, Kang JH, Kim J, Crous-Bou M, et al. Alcohol consumption and the risk of incident pulmonary embolism in US women and men. J Thromb Haemost JTH. 2018;16(9):1753–62.

6. Hu FB, Sigal RJ, Rich-Edwards JW, Colditz GA, Solomon CG, Willett WC, et al. Walking Compared With Vigorous Physical Activity and Risk of Type 2 Diabetes in Women: A Prospective Study. JAMA. 1999;282(15):1433–1439.

7. US Department of Health and Human Services. 2008 physical activity guidelines for Americans [Internet]. 2008 [cited 2020 May 25]. Available from: https://health.gov/our-work/physical-activity/previous-guidelines/2008-physical-activity-guidelines

8. Chiuve SE, Fung TT, Rimm EB, Hu FB, Mccullough ML, Wang M, et al. Alternative dietary indices both strongly predict risk of chronic disease.(Nutrition and Disease)(Author abstract)(Report). J Nutr. 2012;142(6):1009–18.

9. Rimm EB, Stampfer MJ, Colditz GA, Chute CG, Litin LB, Willett WC. Validity of Self-Reported Waist and Hip Circumferences in Men and Women. Epidemiology. 1990;1(6):466–73.

10. World Health Organization. Physical status: the use and interpretation of anthropometry. Report of a WHO Expert Committee. World Health Organ Tech Rep Ser. 1995;854:1–452.

**Supplementary Figure 1: Timing of variable assessment**

| **Supplementary Table S1. Participant characteristics in the full sample (The Nurses’ Health Study II 2001 to 2011, 2013 or 2015 Questionnaire Wave, N=54,703)** | | |
| --- | --- | --- |
| **Questionnaire wave** | | **Mean (SD) or %** |
| **Exposures** (2008 Supplementary Survey) | |  |
| Forgiveness of others | 2008 |  |
| Never/seldom |  | 10.69 |
| Often |  | 52.73 |
| Always/almost always |  | 36.58 |
|  |  |  |
| **Outcomes** (wave 2011, 2013 or 2015) |  |  |
| Psychological well-being |  |  |
| Positive affect (range: 0-3) | 2013 | 2.16 (0.72) |
| Social integration (range: 0-12) | 2013 | 6.44 (2.30) |
| Psychological distress (outcomes) |  |  |
| Depressive symptoms (range: 0-30) | 2013 | 5.84 (4.60) |
| Depression diagnosis | 2015 | 9.87 |
| Anxiety symptoms (range: 0-21) | 2013 | 2.88 (3.42) |
| Hopelessness (range: 0-3) | 2013 | 0.95 (0.96) |
| Loneliness (range: 0-3) | 2013 | 0.47 (0.72) |
| Health behaviors |  |  |
| Heavy drinking | 2011 | 4.51 |
| Current cigarette smoking | 2015 | 4.04 |
| Frequent physical activity | 2013 | 75.48 |
| Preventive healthcare use | 2015 | 89.29 |
| Dietary quality (range: 19.09 to 105.94) | 2011 | 64.53 (13.03) |
| Physical health |  |  |
| All-cause mortality | 2015 | 1.37 |
| No. of physical health problems (range: 0-5) | 2015 | 0.83 (0.73) |
| Diabetes | 2015 | 8.73 |
| Stroke | 2015 | 2.18 |
| Heart Disease | 2013 | 1.68 |
| Cancer | 2015 | 12.45 |
| Overweight/obesity | 2015 | 59.13 |
|  |  |  |
| **Covariates** (wave 2008 or prior) |  |  |
| Sociodemographic characteristics |  |  |
| Age, in years (range: 43-64) | 2008 | 53.37 (4.65) |
| Non-Hispanic White, % | 2005 | 95.75 |
| Married, % | 2005 | 81.41 |
| Geographic region, % | 2007 |  |
| Northeast |  | 31.94 |
| Midwest |  | 32.89 |
| South |  | 18.79 |
| West |  | 16.37 |
| Subjective SES in the U.S. (range:1-10) | 2001 | 7.11 (1.32) |
| Subjective SES in the community (range:1-10) | 2001 | 6.93 (1.58) |
| Pretax household income | 2001 |  |
| <$50,000 |  | 15.74 |
| $50,000–$74,999 |  | 27.33 |
| $75,000–$99,999 |  | 21.38 |
| ≥$100,000 |  | 35.54 |
| Census tract median income, % | 2001 |  |
| <$50,000 |  | 25.67 |
| $50,000–$74,999 |  | 49.01 |
| $75,000–$99,999 |  | 19.06 |
| ≥$100,000 |  | 6.27 |
| Census tract college education rate (range:0-88%) | 2001 | 0.32 (0.16) |
| Currently employed, % | 2001 | 88.78 |
| Childhood abuse victimization (range: 0-5) | 2001 | 1.76 (1.50) |
| Rotating night shift work (over past 2 years), % | 2005 |  |
| None |  | 92.08 |
| 1-9 months |  | 3.39 |
| 10-19 months |  | 1.30 |
| 20+ months |  | 3.23 |
| Religious service attendance, % | 2008 |  |
| Never |  | 23.77 |
| Less than once/week |  | 35.76 |
| At least once/week |  | 40.48 |
| Number of close friends (range: 0-5) | 2008 | 1.73 (0.66) |
| Prior health status or prior health behaviors |  |  |
| Prior positive affect (range: 0-3) | 2008 | 2.10 (0.77) |
| Prior depressive symptoms (range: 0-30) | 2008 | 6.05 (5.01) |
| Prior depression diagnosis, % | 2007 | 15.15 |
| Prior anxiety symptoms (range: 0-15) | 2005 | 2.43 (2.22) |
| Prior hopelessness (range: 0-3) | 2008 | 0.86 (0.92) |
| Prior alcohol intake, % | 2007 |  |
| 0 g/day |  | 33.58 |
| 0.1-9.9 g/day |  | 44.53 |
| 10.0-29.9 g/day |  | 17.82 |
| 30+ g/day |  | 4.07 |
| Prior cigarette smoking, % | 2007 |  |
| never smoker |  | 65.86 |
| former smoker |  | 28.11 |
| current smoker 1-14/d |  | 3.34 |
| current smoker 15-24/day |  | 2.01 |
| current smoker >=25/day |  | 0.68 |
| Prior body max index (kg/m^2^), % | 2007 |  |
| <20 |  | 5.47 |
| 20-24.9 |  | 38.10 |
| 25-29.9 |  | 29.53 |
| 30-34.9 |  | 15.14 |
| 35+ |  | 11.76 |
| Prior physical activity (METS score), % | 2005 |  |
| <3 |  | 15.98 |
| 3-8.9 |  | 18.94 |
| 9-17.9 |  | 20.65 |
| 18-26.9 |  | 14.06 |
| >=27 |  | 30.37 |
| Prior dietary quality (AHEI score), % | 2007 |  |
| Bottom tertile |  | 32.31 |
| Middle tertile |  | 33.33 |
| Top tertile |  | 34.36 |
| Prior preventive healthcare use, % | 2007 | 85.28 |
| Diabetes, % | 2008 | 4.62 |
| CHD, % | 2008 | 1.14 |
| Stroke, % | 2008 | 1.34 |
| Cancer, % | 2008 | 6.56 |
| Postmenopausal status, % | 2007 | 60.65 |
| Replacement Hormone use, % | 2007 | 14.42 |

**Supplementary Table S2. Sensitivity analysis on forgiveness of others and subsequent health and well-being using complete-case analyses (The Nurses’ Health Study II 2008 supplementary survey to 2011, 2013 or 2015 questionnaire wave, N=36,749 to 41,742^a^)**

|  | **Forgiveness of others** ^b^ | | | | | | | | |
| --- | --- | --- | --- | --- | --- | --- | --- | --- | --- |
|  | Often vs. Never/seldom | | | |  | Always/almost always vs. Never/seldom | | | |
| Health and well-being outcomes | RR^c^ | β^d^ | 95% CI | P-value  threshold |  | RR^c^ | β^d^ | 95% CI | P-value  threshold |
| **Psychosocial Well-being** |  |  |  |  |  |  |  |  |  |
| Positive affect |  | 0.09 | 0.06, 0.12 | <.0026^e^ |  |  | 0.19 | 0.16, 0.22 | <.0026^e^ |
| Social integration |  | 0.08 | 0.05, 0.10 | <.0026^e^ |  |  | 0.14 | 0.11, 0.16 | <.0026^e^ |
| **Psychological Distress** |  |  |  |  |  |  |  |  |  |
| Depression diagnosis | 0.94 |  | 0.86, 1.03 |  |  | 0.90 |  | 0.81, 1.00 | <.05 |
| Depressive symptoms |  | -0.08 | -0.11, -0.06 | <.0026^e^ |  |  | -0.16 | -0.19, -0.13 | <.0026^e^ |
| Anxiety symptoms |  | -0.11 | -0.14, -0.08 | <.0026^e^ |  |  | -0.17 | -0.21, -0.14 | <.0026^e^ |
| Hopelessness |  | -0.10 | -0.13, -0.07 | <.0026^e^ |  |  | -0.18 | -0.22, -0.15 | <.0026^e^ |
| Loneliness |  | -0.07 | -0.11, -0.04 | <.0026^e^ |  |  | -0.12 | -0.15, -0.08 | <.0026^e^ |
| **Health Behaviors** |  |  |  |  |  |  |  |  |  |
| Heavy drinking | 1.06 |  | 0.88, 1.29 |  |  | 1.00 |  | 0.81, 1.24 |  |
| Current cigarette smoking | 1.08 |  | 0.87, 1.34 |  |  | 1.16 |  | 0.92, 1.47 |  |
| Frequent physical activity | 1.00 |  | 0.96, 1.04 |  |  | 1.01 |  | 0.96, 1.05 |  |
| Preventive healthcare use | 1.01 |  | 0.97, 1.05 |  |  | 1.00 |  | 0.97, 1.04 |  |
| Dietary quality |  | 0.00 | -0.03, 0.02 |  |  |  | 0.03 | -0.00, 0.06 |  |
| **Physical Health** |  |  |  |  |  |  |  |  |  |
| All-cause mortality | 1.06 |  | 0.79, 1.41 |  |  | 1.23 |  | 0.91, 1.67 |  |
| No. of physical health problems |  | 0.01 | -0.01, 0.03 |  |  |  | 0.00 | -0.03, 0.02 |  |
| Diabetes | 0.98 |  | 0.83, 1.17 |  |  | 1.00 |  | 0.83, 1.21 |  |
| Stroke | 1.30 |  | 0.86, 1.96 |  |  | 1.26 |  | 0.81, 1.95 |  |
| Heart Disease | 0.91 |  | 0.57, 1.46 |  |  | 1.28 |  | 0.78, 2.11 |  |
| Cancer | 0.99 |  | 0.90, 1.09 |  |  | 0.98 |  | 0.90, 1.09 |  |
| Overweight/obesity | 1.01 |  | 0.96, 1.05 |  |  | 0.99 |  | 0.94, 1.03 |  |

Abbreviations: RR, risk ratio; CI, confidence interval.

^a^ The full analytic sample was restricted to those who responded to the Nurses’ Health Study II 2008 supplementary survey in which the exposure variable forgiveness was assessed and had data on forgiveness, all covariates and the outcome variable under investigation.

^b^ A set of generalized estimating equations were used to regress each outcome on forgiveness separately. All models controlled for participants’ age, race, marital status, geographic region, childhood abuse, socioeconomic status (subjective SES, census tract college education rate, and census tract median income), employment status, night shift work schedule, religious service attendance, number of close friends, prior health status or health behaviors (prior depressive symptoms, depression diagnosis, anxiety symptoms, hopelessness, positive affect, dietary quality, body mass index, smoking, alcohol intake, physical activity, preventive healthcare use, postmenopausal status, menopausal hormone therapy use, history of diabetes, heart diseases, stroke, and cancer).

^c^ The effect estimates for the outcomes of heavy drinking, current smoking, mortality, diabetes, heart diseases, stroke and cancer were odds ratio. These outcomes were rare [prevalence<10%], so the odds ratio would approximate RR. Effect estimates for other dichotomized outcomes were RR.

^d^ All continuous outcomes were standardized (mean=0, standard deviation=1), and β was the standardized effect size.

^e^ p<0.05 after Bonferroni correction (the p value cutoff for Bonferroni correction is p=0.05/19 outcomes=0.0026)

**Supplementary Table S3. Sensitivity analysis on forgiveness of others and incident physical health problems using complete-case analyses (The Nurses’ Health Study II 2008 supplementary survey to 2015 questionnaire wave ^a^)**

|  |  | **Forgiveness of others** ^b^ | | | | | | |
| --- | --- | --- | --- | --- | --- | --- | --- | --- |
|  |  | Often vs. Never/seldom | | | |  | Always/almost always  vs. Never/seldom | |
| Incident health outcomes | N | RR^c^ | 95% CI | P-value | | RR^c^ | 95% CI | P-value |
| Diabetes | 38,281 | 0.98 | 0.83, 1.17 | 0.853 |  | 1.00 | 0.83, 1.21 | 0.995 |
| Stroke | 39,478 | 1.30 | 0.86, 1.96 | 0.209 |  | 1.26 | 0.81, 1.95 | 0.302 |
| Heart Disease | 39,953 | 0.91 | 0.57, 1.46 | 0.704 |  | 1.28 | 0.78, 2.11 | 0.329 |
| Cancer | 37,598 | 0.98 | 0.85, 1.13 | 0.771 |  | 0.96 | 0.83, 1.12 | 0.613 |
| Overweight/obesity | 17,356 | 0.99 | 0.88, 1.11 | 0.809 |  | 0.94 | 0.83, 1.07 | 0.342 |
|  |  |  |  |  |  |  |  |  |

Abbreviations: RR, risk ratio; CI, confidence interval.

^a^ The full analytic sample was restricted to those who responded to the Nurses’ Health Study II 2008 supplementary survey in which the exposure variable forgiveness was assessed and had data on forgiveness, all covariates and the outcome variable under investigation. Participants with the corresponding physical health problem at baseline were removed from each analysis, so that the analyses were examining forgiveness in relation to incidence of each physical health problem.

^b^ A set of regression models were used to regress each outcome on forgiveness separately. All models controlled for participants’ age, race, marital status, geographic region, childhood abuse, socioeconomic status (subjective SES, census tract college education rate, and census tract median income), employment status, night shift work schedule, religious service attendance, number of close friends, prior health status or health behaviors (prior depressive symptoms, depression diagnosis, anxiety symptoms, hopelessness, positive affect, dietary quality, smoking, alcohol intake, physical activity, preventive healthcare use, postmenopausal status, and menopausal hormone therapy use). The following baseline characteristics were also either adjusted for as covariates or participants with these conditions at baseline were removed from the analyses in each model, depending on the outcome under investigation: body mass index, history of diabetes, history of heart diseases, history of stroke, and history of cancer.

^c^ The effect estimates for the outcomes of diabetes, heart diseases and stroke were odds ratio. These outcomes were rare [prevalence<10%], so the odds ratio would approximate RR. Effect estimates for cancer and overweight/obesity were RR

**Supplementary Table S4. Forgiveness of others and subsequent health and well-being, stratified by history of exposure to traumatic events that likely involved offenses inflicted by others (The Nurses’ Health Study II 2008 supplementary survey to 2011, 2013 or 2015 questionnaire wave, N=54,703^a^)**

|  | **Forgiveness of others**  (always/almost always vs. never/seldom) ^b^ | | | | | | | | |
| --- | --- | --- | --- | --- | --- | --- | --- | --- | --- |
|  | **Trauma-exposed (n=26,297)** | | | |  | **Trauma non-exposed** **(n=28,406)** | | | |
| Health and well-being outcomes | RR^c^ | β^d^ | 95% CI | P-value  threshold |  | RR^c^ | β^d^ | 95% CI | P-value  threshold |
| **Psychosocial Well-being** |  |  |  |  |  |  |  |  |  |
| Positive affect |  | 0.18 | 0.15, 0.22 | <.0026^e^ |  |  | 0.18 | 0.14, 0.22 | <.0026^e^ |
| Social integration |  | 0.14 | 0.11, 0.17 | <.0026^e^ |  |  | 0.15 | 0.12, 0.19 | <.0026^e^ |
| **Psychological Distress** |  |  |  |  |  |  |  |  |  |
| Depression diagnosis | 0.92 |  | 0.83, 1.02 |  |  | 0.90 |  | 0.76, 1.06 |  |
| Depressive symptoms |  | -0.18 | -0.21, -0.14 | <.0026^e^ |  |  | -0.15 | -0.18, -0.11 | <.0026^e^ |
| Anxiety symptoms |  | -0.13 | -0.17, -0.09 | <.0026^e^ |  |  | -0.09 | -0.14, -0.05 | <.0026^e^ |
| Hopelessness |  | -0.17 | -0.20, -0.13 | <.0026^e^ |  |  | -0.17 | -0.22, -0.12 | <.0026^e^ |
| Loneliness |  | -0.15 | -0.19, -0.11 | <.0026^e^ |  |  | -0.09 | -0.13, -0.05 | <.0026^e^ |
| **Health Behaviors** |  |  |  |  |  |  |  |  |  |
| Heavy drinking | 0.87 |  | 0.65, 1.16 |  |  | 1.26 |  | 0.94, 1.70 |  |
| Current cigarette smoking | 1.08 |  | 0.84, 1.39 |  |  | 0.85 |  | 0.61, 1.20 |  |
| Frequent physical activity | 1.01 |  | 0.96, 1.06 |  |  | 1.01 |  | 0.96, 1.07 |  |
| Preventive healthcare use | 1.00 |  | 0.96, 1.04 |  |  | 1.01 |  | 0.96, 1.06 |  |
| Dietary quality |  | 0.06 | 0.02, 0.10 | <.01 |  |  | 0.02 | -0.01, 0.06 |  |
| **Physical Health** |  |  |  |  |  |  |  |  |  |
| All-cause mortality | 1.22 |  | 0.86, 1.75 |  |  | 1.12 |  | 0.75, 1.67 |  |
| No. of physical health problems |  | -0.01 | -0.04, 0.02 |  |  |  | 0.00 | -0.04, 0.03 |  |
| Diabetes | 0.94 |  | 0.76, 1.16 |  |  | 0.98 |  | 0.75, 1.27 |  |
| Stroke | 1.21 |  | 0.79, 1.84 |  |  | 1.18 |  | 0.60, 2.32 |  |
| Heart Disease | 1.35 |  | 0.77, 2.39 |  |  | 0.92 |  | 0.47, 1.80 |  |
| Cancer | 1.04 |  | 0.92, 1.17 |  |  | 0.93 |  | 0.81, 1.06 |  |
| Overweight/obesity | 0.98 |  | 0.92, 1.03 |  |  | 1.01 |  | 0.95, 1.08 |  |

Abbreviations: RR, risk ratio; CI, confidence interval.

^a^ The full analytic sample was restricted to those who responded to the Nurses’ Health Study II 2008 supplementary survey in which the exposure variable forgiveness was assessed. Multiple imputation was performed to impute missing data on all variables.

^b^ A set of generalized estimating equations were used to regress each outcome on forgiveness separately. All models controlled for participants’ age, race, marital status, geographic region, childhood abuse, socioeconomic status (subjective SES, household income, census tract college education rate, and census tract median income), employment status, night shift work schedule, religious service attendance, number of close friends, prior health status or health behaviors (prior depressive symptoms, depression diagnosis, anxiety symptoms, hopelessness, positive affect, dietary quality, body mass index, smoking, alcohol intake, physical activity, preventive healthcare use, postmenopausal status, menopausal hormone therapy use, history of diabetes, heart diseases, stroke, and cancer).

^c^ The effect estimates for the outcomes of heavy drinking, current smoking, mortality, diabetes, heart diseases, stroke and cancer were odds ratio. These outcomes were rare [prevalence<10%], so the odds ratio would approximate RR. Effect estimates for other dichotomized outcomes were RR.

^d^ All continuous outcomes were standardized (mean=0, standard deviation=1), and β was the standardized effect size

^e^ p<0.05 after Bonferroni correction (the p value cutoff for Bonferroni correction is p=0.05/19 outcomes=0.0026).
